# Supplementary material for: MetaQTL: a package of new computational methods for the meta-analysis of QTL mapping experiments
Source: BMC Bioinformatics. 2007 Feb 8;8:49. doi: 10.1186/1471-2105-8-49 (PMC1808479; doi:10.1186/1471-2105-8-49)
Supplement: Additional File 7 — MetaQTL Package : jar file and tutorial. This Zip archive contains both the MetaQTL JAR file and the files of the tutorial. [file 1471-2105-8-49-S7.zip › org.inra.metaqtl/doc/org/metaqtl/class-use/MultiFitResult.html]

Uses of Class org.metaqtl.MultiFitResult


|  |  |  |
| --- | --- | --- |
| **Overview** \| **Package** \| **Class** \| **Use** \| **Tree** \| **Deprecated** \| **Index** \| **Help** | |  |
| PREV   NEXT | **FRAMES**    **NO FRAMES**     **All Classes** |  |


---


## **Uses of Class org.metaqtl.MultiFitResult**

| Packages that use MultiFitResult | |
| --- | --- |
| **org.metaqtl** |  |
| **org.metaqtl.algo** |  |

Uses of MultiFitResult in org.metaqtl

Constructors in org.metaqtl with parameters of type MultiFitResult

`MetaChrom(MultiFitResult result,
ChromCluster chromCluster)`
  
          Get a new instance of `MetaChrom` which is
initialized by using the result
of the merge of the members of a `ChromCluster`.

Uses of MultiFitResult in org.metaqtl.algo

| Methods in org.metaqtl.algo that return MultiFitResult | |
| --- | --- |
| `static MultiFitResult` | `SVDAlgorithm.SVDMFit(double[][] X, double[] y, double[] w, int m, int n)`             Given a set of data points y[1..m] with indiviudal weights w[1..m], use chi2 minimization to determine the coefficients a[1..n] of the fitting function y = X\*a. |

---


|  |  |  |
| --- | --- | --- |
| **Overview** \| **Package** \| **Class** \| **Use** \| **Tree** \| **Deprecated** \| **Index** \| **Help** | |  |
| PREV   NEXT | **FRAMES**    **NO FRAMES**     **All Classes** |  |


---
